# Oscillations of a soft viscoelastic drop

**DOI:** 10.1038/s41526-021-00169-1

**Published:** 2021-11-02

**Authors:** Saiful I. Tamim, Joshua B. Bostwick

**Affiliations:** Department of Mechanical Engineering, Clemson University, Clemson, SC 29634 USA

**Keywords:** Fluid dynamics, Gels and hydrogels, Rheology, Polymers

## Abstract

A soft viscoelastic drop has dynamics governed by the balance between surface tension, viscosity, and elasticity, with the material rheology often being frequency dependent, which are utilized in bioprinting technologies for tissue engineering and drop-deposition processes for splash suppression. We study the free and forced oscillations of a soft viscoelastic drop deriving (1) the dispersion relationship for free oscillations, and (2) the frequency response for forced oscillations, of a soft material with arbitrary rheology. We then restrict our analysis to the classical cases of a Kelvin–Voigt and Maxwell model, which are relevant to soft gels and polymer fluids, respectively. We compute the complex frequencies, which are characterized by an oscillation frequency and decay rate, as they depend upon the dimensionless elastocapillary and Deborah numbers and map the boundary between regions of underdamped and overdamped motions. We conclude by illustrating how our theoretical predictions for the frequency-response diagram could be used in conjunction with drop-oscillation experiments as a “drop vibration rheometer”, suggesting future experiments using either ultrasonic levitation or a microgravity environment.

## Introduction

There is a long history of experimental studies of drop oscillations in ultrasonic levitation^1^ and microgravity^2,3^. These oscillations are governed by the length and timescales of the drop that correspond to the different forces like capillarity, viscosity, and elasticity. The choice of scales where these effects dominate is limited in most cases due to the additional gravitational effect being present in experiments. The advantages of the microgravity environment are the large length and timescales not accessible under terrestrial conditions, as well as the high degree of drop sphericity that can be achieved. This allows for a direct comparison to the classical theory of Lord Rayleigh^4^ who showed that an inviscid spherical drop of radius *R* will oscillate about its equilibrium shape with characteristic frequency,

1 $$\omega =\sqrt{l(l-1)(l+2)\frac{\sigma }{\rho {R}^{3}}},$$

and mode shape given by the spherical harmonic ${Y}_{l}^{m}$. Here, *ω* is angular frequency in rad/s, *ρ* is the liquid density, *σ* the liquid/gas surface tension, *l* the polar mode number, and *m* the azimuthal mode number. Note that the Rayleigh spectrum (1) is degenerate with respect to the azimuthal mode number *m*. Extensions to this basic model include, but are not limited to, the effect of viscosity^5^, large-amplitude deformation^6^, and wetting^7^. Relatively fewer models have been proposed to study the viscoelastic effects^8,9^. In this paper, we fill this critical gap in the literature by developing a comprehensive theory of the oscillations of a soft viscoelastic drop.

Dynamic drop response of viscous Newtonian fluids is seen in applications such as inkjet printing^10^ and spray cooling^11^. Adding a polymer to a simple viscous fluid can induce a non-Newtonian viscoelastic response that can greatly affect the dynamics of, e.g., pinch-off, surface impact, spreading, and bouncing^12–14^. Viscoelastic fluid droplets, in particular, are prominent in inkjet printing^15,16^, drop deposition^17^, and spray atomization^18^. Similar viscoelastic effects are seen in soft solids like hydrogels, which have cross-linked polymer networks with tunable elasticity^19,20^, and are widely used as biocompatible materials in rapid prototyping technologies^21,22^ and drug-delivery systems^23^. Both polymer fluids and soft gels are viscoelastic materials with both a viscosity and elasticity, both of which can have a complex dependence on frequency defining the rheology of the material through a storage (elasticity) and loss (viscosity) modulus.

The role of viscosity in free drop oscillations is to (1) decrease the natural frequency and (2) introduce a nontrivial decay rate^24,25^. Recently, the inviscid Rayleigh drop theory was extended to account for the nontrivial elasticity found in many soft hydrogels through a linear elastic model^26^. The dynamics of soft gels are described by the elastocapillary number Γ = *σ*/*μR*, which is the balance between the capillary ${t}_{c}=\sqrt{\rho {R}^{3}/\sigma }$ and elastic ${t}_{e}=\sqrt{\rho {R}^{2}/\mu }$ time scales^27^. Here, *μ* is the shear modulus that is a constant property of linear elastic materials. Elastocapillary effects have been observed in many of the classical interfacial instabilities of hydrodynamics, but at soft solid interfaces^28–31^. Soft materials with a complex rheology exhibit an additional viscoelastic timescale *τ* over which viscous dissipation occurs. For fluids, this refers to the relaxation time under constant stress, and for solids to the creep time under constant strain. The balance of *τ* with either *t*_*c*_ or *t*_*e*_ gives rise to the Deborah number^32^. Prior work has focused on limiting values of *τ*, but here we focus more deeply on the effect of the viscoelastic timescale on the oscillation of drops with complex rheologies. In particular, we focus on the Kelvin–Voigt model as a proxy for viscoelastic “solids” and the Maxwell model as a proxy for viscoelastic “fluids”.

Previous studies on surface waves in viscoelastic materials have predicted complex eigenfrequencies with the real part corresponding to the oscillation frequency and imaginary part the decay rate of oscillation^33–35^. Naturally, the purely elastic limit has no decay rate. Pioneering works by Trinh et al.^1^ have shown the potential of acoustic levitation to create a low-gravity environment for a free viscous drop, from which oscillations can occur and the viscosity can be inferred from the experimentally observed dynamics. The main advantage of this approach is that it is containerless and avoids the effect of wetting, as seen in the oscillations of a sessile drop^36,37^. Recent works have utilized drop oscillations to measure the material properties of viscoelastic materials^38,39^. Unfortunately, to use these techniques to more accurately measure material properties requires the development of more sophisticated theoretical models that account for viscoelastic effects for an arbitrary material rheology.

In this paper, we derive the dispersion relationship and frequency-response function for an oscillating viscoelastic drop with arbitrary rheology. We illustrate the utility of our solution using the classical rheological models of Kelvin–Voigt and Maxwell, which are relevant to soft gels and polymer fluids, respectively. These simple models are based on an exponential temporal response characterized by a single viscous timescale, which when nondimensionalized, gives rise to a Deborah number that defines the viscoelastic time scale. Many industrial soft materials such as biopolymers and foods exhibit a more complex power-law response^40–43^, which we also investigate using fractional variants of the Kelvin–Voigt and Maxwell models^44–46^. In general, the motions can be underdamped or overdamped, depending upon the Deborah number, and we map out these regions in the parameter space. We conclude with a discussion of how our theoretical model could be used in conjunction with experiments as a “drop vibration rheometer” to measure the material properties of soft viscoelastic materials.

## Methods

### Field equations

**Fig. 1:**
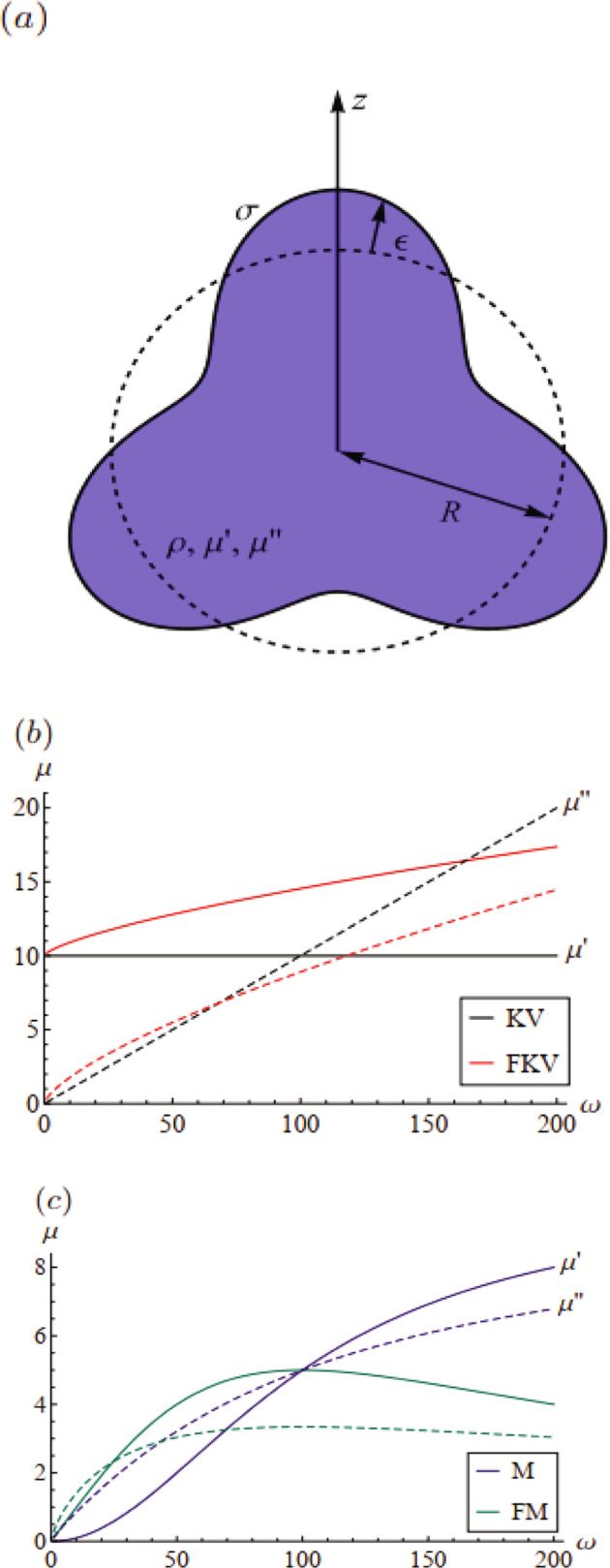
Definition sketch and material rheologies. **a** Shape of the deformed drop with rheology defined by the frequency-dependent complex modulus $\mu (\omega )=\mu ^{\prime} (\omega )+i\mu ^{\prime\prime} (\omega )$. Storage modulus $\mu ^{\prime}$ (solid line) and loss modulus *μ″* (dashed line) are shown for **b** Kelvin–Voigt (KV) and **c** Maxwell (M) models with their respective fractional counterparts.

Consider the spherical drop with equilibrium radius *R* shown in Fig. 1a. The drop is incompressible and has material properties defined by the density *ρ*, complex modulus *μ*, and surface tension *σ*. The displacement field is expressed in three-dimensional spherical coordinates,

2 $${{{\bf{U}}}}={U}_{r}(r,\theta ,\varphi ,t){\hat{e}}_{r}+{U}_{\theta }(r,\theta ,\varphi ,t){\hat{e}}_{\theta }+{U}_{\varphi }(r,\theta ,\varphi ,t){\hat{e}}_{\varphi }.$$

The drop is assumed to behave as a linear viscoelastic material in which the stress field **T**(***x***, *t*) is related to the strain field ***ε***(***x***, *t*) through the following relationship^47^:

3 $${{{\bf{T}}}}(t)=-p{{{\bf{I}}}}+2\int\nolimits_{-\infty }^{t}G(t-t^{\prime} )\frac{\partial {{{\boldsymbol{\varepsilon }}}}(t^{\prime} )}{\partial t^{\prime} }dt^{\prime} ,$$

Here, *G*(*t*) is the relaxation function and *p* is the pressure. The strain field *ε*_*ij*_(**x**, *t*) is related to the displacement **U**(**x**, *t*),

4 $${{{\boldsymbol{\varepsilon }}}}=\frac{1}{2}\left(\nabla {{{\bf{U}}}}+\nabla {{{{\bf{U}}}}}^{T}\right),$$

which satisfies the dynamic equilibrium and incompressibility equations,

5a $$\nabla \cdot {{{\bf{T}}}}=\rho \frac{{\partial }^{2}{{{\bf{U}}}}}{\partial {t}^{2}},$$

5b $$\nabla \cdot {{{\bf{U}}}}=0.$$

### Frequency domain

Normal modes **U**(**x**, *t*) = **u**(**x**)*e*^*iωt*^ are assumed with frequency *ω* and the field equations are transformed into the frequency domain *ω* by the Fourier transform,

6 $$\tilde{f}(\omega )=\int\nolimits_{-\infty }^{\infty }f(t){e}^{i\omega t}.$$

This results in the following time-independent field equation,

7 $$-\nabla p+\tilde{\mu }{\nabla }^{2}{{{\bf{u}}}}=-\rho {\omega }^{2}{{{\bf{u}}}},$$

subject to the following boundary conditions at the drop interface *r* = *R*:

8 $${T}_{rr}=\frac{\sigma }{{R}^{2}}\left({\nabla }_{| | }^{2}+2\right){u}_{r}(R),$$

9 $${T}_{r\theta }=0,$$

10 $${T}_{r\varphi }=0,$$

which correspond to continuity of stress. Note that Eq. (8) is the Young–Laplace equation with ${\nabla }_{| | }^{2}$ the surface Laplacian.

Here we define the frequency-dependent complex modulus,

11 $$\tilde{\mu }(\omega )=i\omega \int\nolimits_{0}^{\infty }G(t){e}^{-i\omega t}dt,$$

which can be written as $\tilde{\mu }(\omega )=\mu ^{\prime} (\omega )+i\mu ^{\prime\prime} (\omega )$ with $\mu ^{\prime} (\omega )$ the storage modulus and *μ″*(*ω*) the loss modulus. The form of the complex modulus $\tilde{\mu }(\omega )$ depends on the particular rheology of the material. We illustrate our solution method on two classic rheological models of viscoelasticity, the Kelvin–Voigt model and the Maxwell model. These models are the limiting cases of their fractional variants that are relevant to soft polymeric materials. This is shown in Table 1 and plotted in Fig. 1b. Here, the Kelvin–Voigt (KV) models have nonzero shear modulus *μ*_*o*_ at zero frequency and therefore are more representative of “solid-like” materials like gels. The Maxwell (M) models have zero-loss modulus at high frequency and are applicable to “fluid-like” materials, such as polymer solutions. Both models have a material response modeled as a combination of a single spring and dashpot, and therefore are characterized by a single viscoelastic timescale, *τ*_*s*_ and *τ*_*f*_ for Kelvin–Voigt and Maxwell, respectively.

### Displacement potentials

We construct a solution for the displacement field as a combination of scalar potentials (Φ, *Q*, *S*),

12 $${{{\bf{u}}}}=\nabla {{\Phi }}+\nabla \times (rQ{\hat{e}}_{r})+\nabla \times \nabla \times (rS{\hat{e}}_{r}).$$

This decomposition yields the pressure field *p* = *ρω*^2^Φ + *P*_*o*_(*t*), where *P*_*o*_ is a harmonic function of time, which can refer to an external pressure, which we will discuss shortly. Applying (12) to (7) results in a set of decoupled wave equations,

13 $${\nabla }^{2}{{\Phi }}=0,\quad {\nabla }^{2}Q+{\beta }^{2}Q=0,\quad {\nabla }^{2}S+{\beta }^{2}S=0,$$

with $\beta =\omega r\sqrt{\frac{\rho }{\tilde{\mu }}}$. The resulting motions can be further decomposed into shape change (Φ, *S*) and torsional (*Q*) modes, similar to the case of the purely elastic sphere^48^. The general solution for the displacement potentials can be expressed using a series expansion with spherical harmonic basis ${Y}_{l}^{m}(\theta ,\phi )$,

14 $$\begin{array}{lll}{{\Phi }}&=&\mathop{\sum}\limits_{l,m}{A}_{lm}{r}^{l}{Y}_{l}^{m}(\theta ,\phi ),\quad Q=\mathop{\sum}\limits_{l,m}{B}_{lm}{j}_{l}(\beta r){Y}_{l}^{m}(\theta ,\phi )\\ S&=&\mathop{\sum}\limits_{l,m}{C}_{lm}{j}_{l}(\beta r){Y}_{l}^{m}(\theta ,\phi ).\end{array}$$

Here, *j*_*l*_ is the spherical Bessel function, *l* = 0, ⋯ , *∞* the polar mode number, and *m* = − *l*, ⋯ , *l* the azimuthal mode number. The unknown constants *A*, *B*, and *C* are determined from the boundary conditions Eqs. (8)–(10), and are given in the Supplementary Note.

### Dispersion relationship

In the absence of an external pressure field *P*_*o*_ = 0, the drop undergoes free oscillations with characteristic frequency determined from the dispersion relationship that results from the solvability condition of Eqs. (8)–(10). Here the dispersion relationship for the torsional modes results from Eq. (10) and the dispersion relationship for the shape-change modes from Eqs. (8) and (9). Our focus is on the shape-change modes with dispersion relationship given by

15 $$\begin{array}{l}\xi \sqrt{\tilde{{{\Gamma }}}}\left(2+{\xi }^{2}\tilde{{{\Gamma }}}+2l-4{l}^{2}-\tilde{{{\Gamma }}}l({l}^{2}+l-2)\right){j}_{l}(\xi \sqrt{\tilde{{{\Gamma }}}})\\ -2\left({\xi }^{2}\tilde{{{\Gamma }}}+l(\tilde{{{\Gamma }}}+2)({l}^{2}+l-2)\right){j}_{l+1}(\xi \sqrt{\tilde{{{\Gamma }}}})=0.\end{array}$$

Here, $\xi ={\omega }_{n}\sqrt{\rho {R}^{3}/\sigma }$ is the scaled eigenfrequency and $\tilde{{{\Gamma }}}=\sigma /\tilde{\mu }R$ the elastocapillary number. The dispersion relation for the torsional modes is given in the supplementary Note.

### Reporting summary

Further information on research design is available in the Nature Research Reporting Summary linked to this article.

## Results

Any of the rheological models given in Table 1 can be applied to Eq. (15) from which we can compute the complex eigenfrequencies *ξ*, where the real part of *ξ* gives the oscillation frequency and the imaginary part of *ξ* gives the decay rate. We note the absence of the azimuthal wavenumber *m* from Eq. (15) implying that the frequency spectrum is degenerate with respect to *m* for any rheological model, like the Rayleigh drop. For this reason, we will restrict ourselves to the axisymmetric (*m* = 0) modes. Unlike the Rayleigh drop, Eq. (15) is a nonlinear dispersion equation, which admits an infinity of roots for the same polar mode *l*, which defines the radial mode number *s*. That is, each motion is defined by the mode-number pair (*l*, *s*). For any *l*, the interface shape is identical, but the internal flow field is distinguished by *s*, as discussed by Tamim & Bostwick^26^ for a purely elastic drop.

**Table 1:** Complex modulus $\tilde{\mu }(\omega )$ for the cases studied here with corresponding high and low frequency limits.

Model	$\tilde{\mu }$	Viscous limit	Elastic limit
KV	*μ*_*o*_(1 + *iωτ*_*s*_)	*ω* = *∞*	*ω* = 0
M	${\mu }_{e}\frac{i\omega {\tau }_{f}}{1+i\omega {\tau }_{f}}$	*ω* = 0	*ω* = *∞*
FKV	${\mu }_{o}\left(1+{(i\omega {\tau }_{s})}^{n}\right)$	*ω* = *∞*	*ω* = 0
FM	${\mu }_{e}\frac{{(i\omega {\tau }_{f})}^{n}}{1+{(i\omega {\tau }_{f})}^{n}}$	*ω* = 0	*ω* = *∞*

### Kelvin–Voigt model (soft solids)

**Fig. 2:**
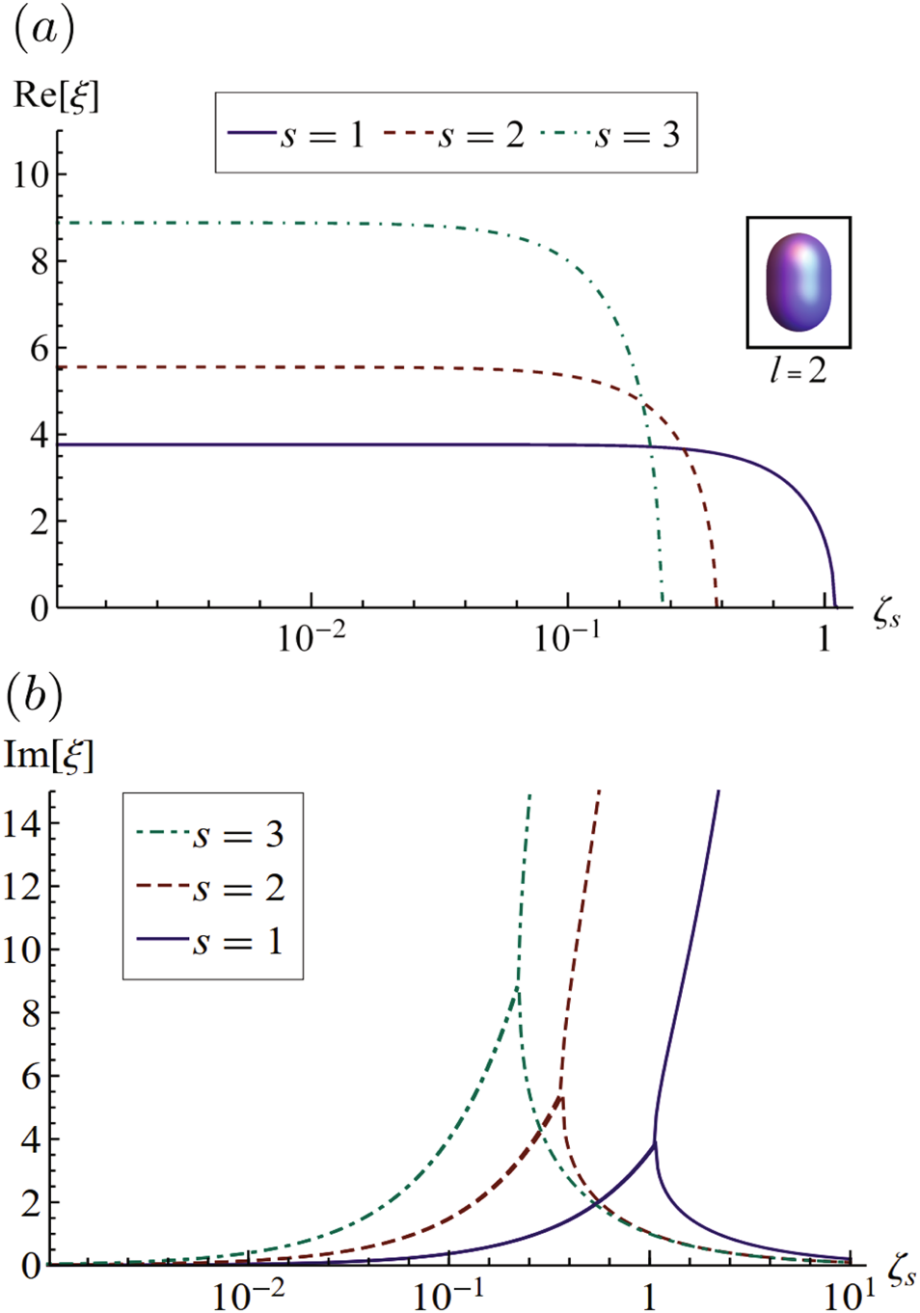
Complex frequency for a Kelvin–Voigt material. **a** Frequency ${{{\rm{Re}}}}[\xi ]$ and **b** decay rate ${{{\rm{Im}}}}[\xi ]$ plotted against the solid Deborah number *ζ*_*s*_, as it depends upon the radial mode number *s*, for *l* = 2, Γ = 1.

We begin with the simplest case of a soft viscoelastic solid, as relevant to gels, the Kelvin–Voigt model. Here, scaling the viscoelastic timescale *τ*_*s*_ with the elastic wave timescale gives rise to the solid Deborah number ${\zeta }_{s}={\tau }_{s}\sqrt{\mu /\rho {R}^{2}}$ with limiting cases corresponding to *ζ*_*s*_ = 0 the purely elastic limit and *ζ*_*s*_ = *∞* the purely viscous limit. In addition, we define the static elastocapillary number ${{\Gamma }}=\frac{\sigma }{{\mu }_{o}R}$, where *μ*_*o*_ is the equilibrium shear modulus. Figure 2 plots the complex frequency *ξ* for *l* = 2 mode against *ζ*_*s*_ for the first three radial modes *s*. In the small *ζ*_*s*_ region, the motion is underdamped and characterized by an oscillation frequency ${{{\rm{Re}}}}[\xi ]$ that is essentially constant with a decay rate ${{{\rm{Im}}}}[\xi ]$ that increases with *ζ*_*s*_. As *ζ*_*s*_ increases, the motion becomes overdamped ${{{\rm{Re}}}}[\xi ]=0$ at a critical value of *ζ*_*s*_, where a bifurcation occurs and beyond which no oscillation is observed. Modes with higher radial mode number *s* become overdamped at lower values of *ζ*_*s*_.

**Fig. 3:**
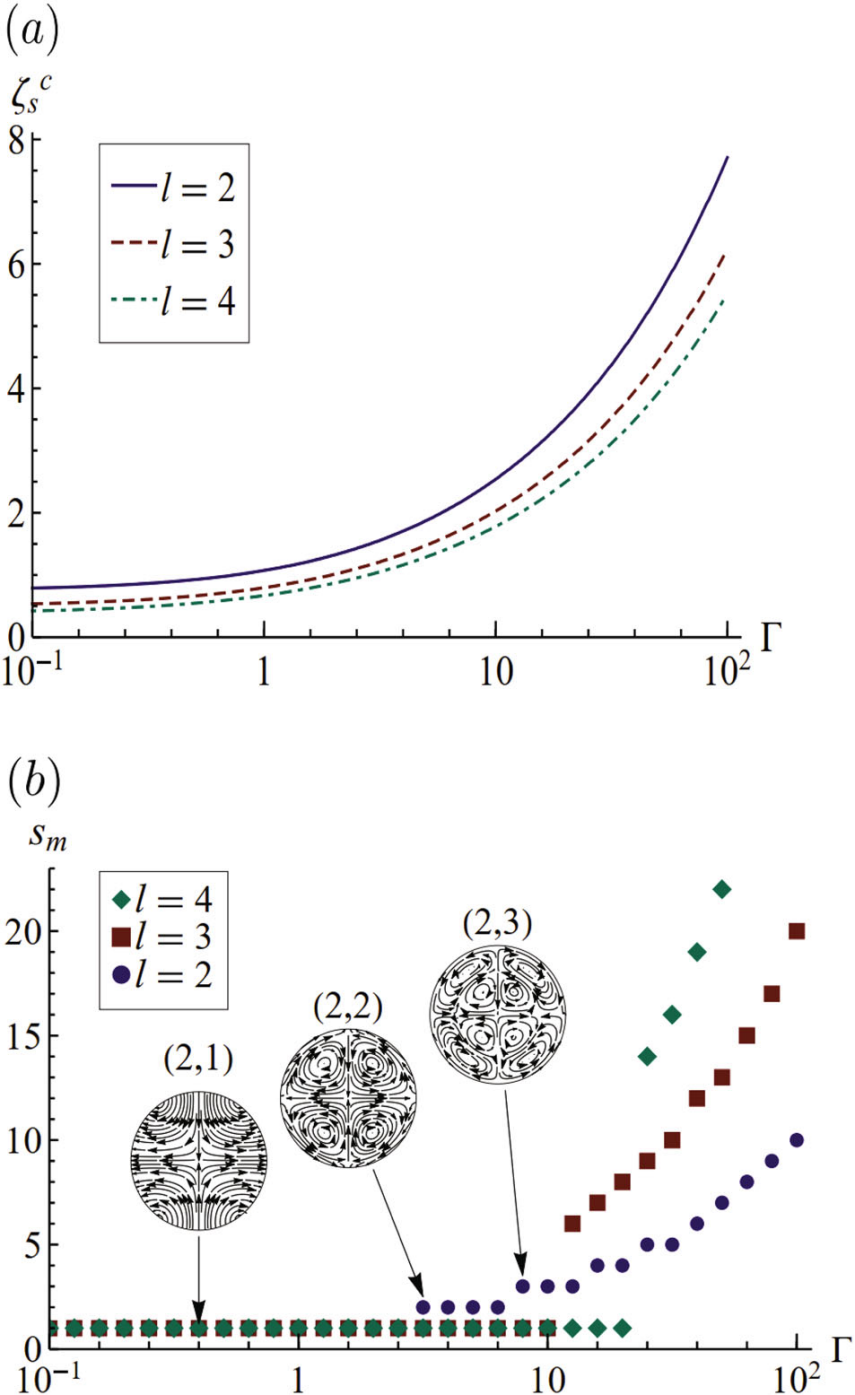
Elastocapillary effects for Kelvin–Voigt material. Plots show **a** critical Deborah number for overdamped motion ${\zeta }_{s}^{c}$ of the least-damped mode *s*_*m*_, as it depends upon *l*, and **b** the radial mode number *s* for the least-damped mode with *ζ*_*s*_ = 0.01, against Γ. The internal flow field for a few modes (*l*, *s*) is shown as an inset.

The least-damped mode is the one with the smallest decay rate ${{{\rm{Im}}}}[\xi ]$ and largest critical Deborah number ${\zeta }_{s}^{c}$ and this is the one most likely to be observed during experiment. For the conditions shown in Fig. 2, this is the *s* = 1 mode, but this is not always the case. Figure 3a plots the critical solid Deborah number ${\zeta }_{s}^{c}$ where all the roots of the dispersion relation (15) are purely imaginary, i.e., overdamped, against the elastocapillary number Γ. Here ${\zeta }_{s}^{c}$ increases with Γ for all polar mode numbers *l*. This shows that when surface-tension effects on the drop are increased, higher magnitude of viscoelasticity is required to cause critical damping. Figure 3b plots the least-damped mode *s*_*m*_ for a given polar mode *l* against Γ. For small surface-tension effects Γ ≪ 1, the *s* = 1 mode is the least-damped mode for all *l*. However, for larger Γ, surface tension becomes significant and higher radial mode numbers *s* begin to show the least amount of damping. These motions are differentiated by their internal flow fields, as shown as insets to Fig. 3b. Higher *s* corresponds to a larger number of vortices in the internal flow fields. This means that increased effect of capillarity causes higher frequency roots to have less damping and be more dominant. This is also consistent with the analysis of Tamim and Bostwick^26^, which shows higher capillary effect causes higher frequency in a given vibration mode.

**Fig. 4:**
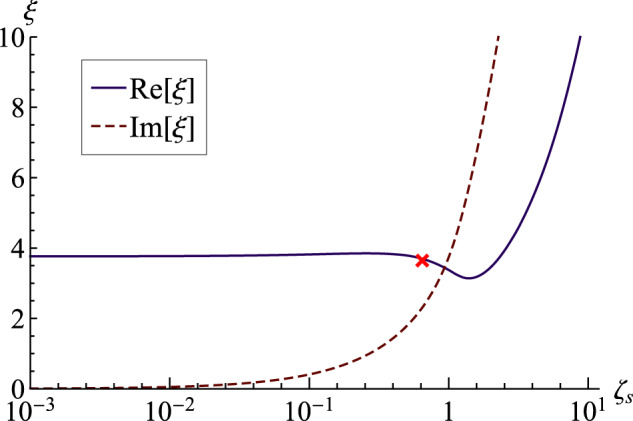
Complex frequency against Deborah number for fractional Kelvin–Voigt material. Re[*ξ*] and Im[*ξ*] plotted against solid Deborah number *ζ*_*s*_ with Γ = 1. The red cross refers to the point where the resonance peak in forced vibration has disappeared.

The fractional Kelvin–Voigt model is characterized by the power-law exponent *n* with limiting case *n* = 1 corresponding to Kelvin–Voigt model. For *n* < 1, there is not always a transition from underdamped to overdamped motion with increasing *ζ*_*s*_. Here, the fractional damping does not produce purely imaginary roots or overdamped motions^49^. Figure 4 plots the complex frequency *ξ* against *ζ*_*s*_ for the (2, 1) mode with *n* = 0.9. Here both the real and imaginary parts of *ξ* are nonzero for all *ζ*_*s*_, implying that there are always underdamped oscillations. This implies that the fractional Kelvin–Voigt model does not predict any overdamped motion. Rossikhin and Shitikova^50^ have also shown that aperiodic modes of decay do not appear in the fractional Kelvin–Voigt model with *n* < 1. Also shown in Fig. 4 is that ${{{\rm{Im}}}}[\xi ]$ monotonically increases with *ζ*_*s*_, whereas ${{{\rm{Re}}}}[\xi ]$ shows a more complex dependence. Here the oscillation frequency ${{{\rm{Re}}}}[\xi ]$ begins to decrease, indicative of the approach to overdamped motion, but instead then increases. Even though the complex frequency predicts free underdamped oscillations, we show later in the forced-oscillation problem that the corresponding resonance peak in the frequency-response diagram can disappear for this mode at a particular value of *ζ*_*s*_. The reason that fractional Kelvin model does not predict purely aperiodic modes is due to the fact that this model is characterized by a so-called “springpot” instead of an ideal dashpot to account for the viscoelastic effect^46^. This springpot shows behavior that is intermediate between a spring and a dashpot, and therefore it always predicts a nonzero oscillation frequency.

### Maxwell model (polymer fluids)

**Fig. 5:**
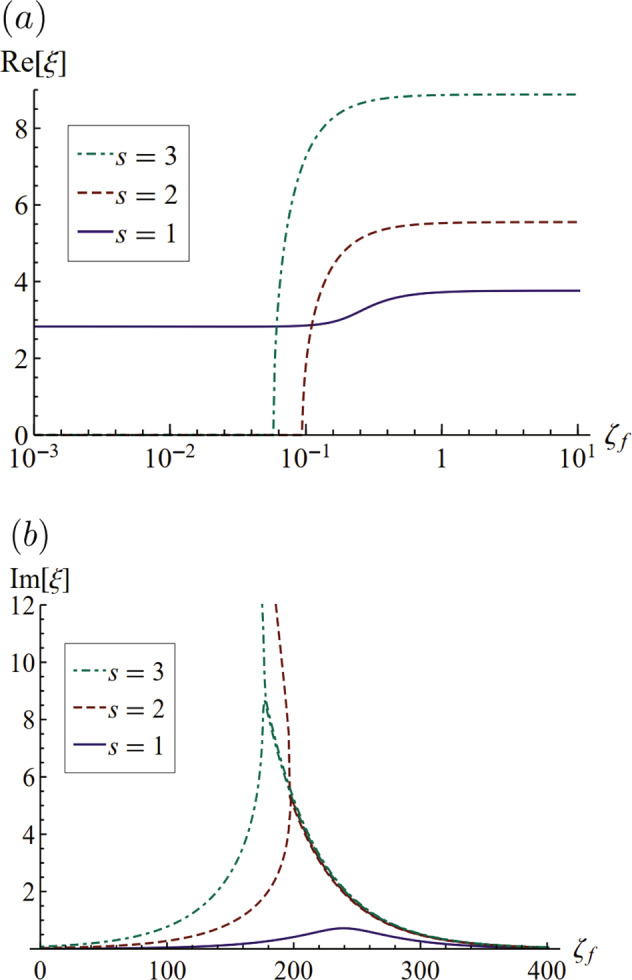
Complex frequency against Deborah number for Maxwell material. **a** plots the frequency ${{{\rm{Re}}}}[\xi ]$ and **b** plots the decay rate ${{{\rm{Im}}}}[\xi ]$, as it depends upon the radial mode number *s*, for *l* = 2, Γ = 1.

Polymeric fluids are well described by the Maxwell model. Here, scaling the relaxation timescale *τ*_*f*_ with the capillary timescale gives rise to the fluid Deborah number ${\zeta }_{f}={\tau }_{f}\sqrt{\sigma /\rho {R}^{3}}$, from which *ζ*_*f*_ = 0 corresponds to viscous fluids and *ζ*_*f*_ = *∞* to purely elastic solids. Figure 5 plots the complex frequency *ξ* against *ζ*_*f*_ for the (2, *s*) mode. Here in the low *ζ*_*f*_ region, there is only one mode with nontrivial oscillation frequency, which corresponds to that of the Rayleigh drop^4^, $\xi =\sqrt{l(l-1)(l+2)}=2.828$. In the small *ζ*_*f*_ region, there are many overdamped modes ${{{\rm{Re}}}}[\xi ]$ and similar to the Kelvin model, these overdamped modes have two rates of decay for the same Deborah number, as shown in Fig. 5b. Here, we find that the *s* = 1 root shows the least amount of damping when compared with the higher-order roots. Similar to the Kelvin model, the order of the least-damped mode in the Maxwell model can also be a function of elastocapillary number Γ. This means that higher Γ will cause higher-order roots to be more dominant. The critical Deborah number *ζ*_*f*_ where this bifurcation occurs marks the boundary between overdamped and underdamped motions for these modes and ${{{\rm{Re}}}}[\xi ]$ plateaus to a constant value marking an underdamped motion. The least-damped *s* = 1 mode also plateaus to a higher frequency in the underdamped region. This is because in the underdamped region, elasticity dominates viscous damping and adds a positive contribution to the oscillation frequency.

**Fig. 6:**
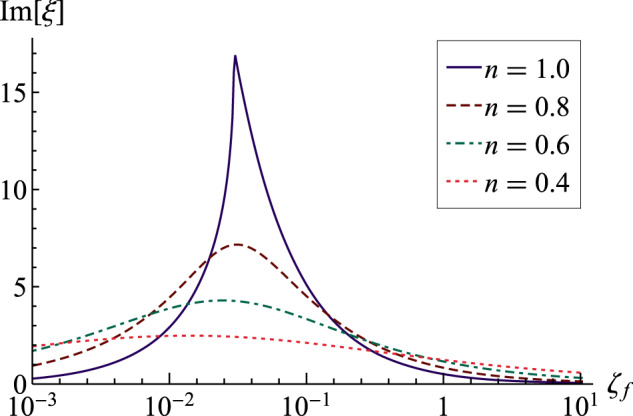
Decay rate against Deborah number for fractional Maxwell model. Im[*ξ*] plotted against Deborah number *ζ*_*f*_ with Γ = 0.1 as it depends upon the power-law exponent *n*.

Similar to the fractional Kelvin model, the fractional Maxwell model also does not predict a critical Deborah number where the frequency transitions between overdamped and underdamped motion. This is shown in Fig. 6, which plots the imaginary part of the frequency ${{{\rm{Im}}}}[\xi ]$ as a function of *ζ*_*f*_ as it depends upon the power-law exponent *n*. Here there is a sharp peak in the decay rate for the limiting case of the Maxwell model *n* = 1, which corresponds to the critical Deborah number *ζ*_*f*_. This demarcation between underdamped and overdamped motions disappears for *n* < 1 and the curves show a maximum that decreases with a further decrease in power-law exponent *n*. This behavior of the fractional Maxwell model was previously observed in the response of the one-dimensional system using a fractional calculus approach^51^.

### Forced oscillations

It is straightforward to adapt our analysis to the forced-oscillation problem in which the drop is driven by an oscillatory pressure with magnitude *P*_*d*_. Here the pressure on the drop becomes *p* = *ρω*^2^Φ + *P*_*d*_*e*^*iωt*^ and the analysis is identical to the free-oscillation case. Applying the general solutions in Eq. (14) to the boundary equations in Eqs. (8, 9) yields an expression for the radial displacement at the drop surface *u*_*r*_(*R*). We can scale the displacement $x={u}_{r}(R)\tilde{\mu }/{P}_{d}R$ and driving frequency ${{\Omega }}=\omega \sqrt{\rho {R}^{3}/\sigma }$, and define $\eta ={{\Omega }}\sqrt{\tilde{{{\Gamma }}}}$. In this case, the scaled displacement is given by

16 $$\begin{array}{ll}x=&\mathop{\sum }\limits_{l=0}^{\infty }\mathop{\sum }\limits_{m=-l}^{l}(2{j}_{l+1}(\eta )-l(\eta {j}_{l}(\eta ))\cdot {Y}_{l}^{m}(\theta ,\phi ))/\\ &\eta {j}_{l}(\eta )({\eta }^{2}+2-4{l}^{2}+2l-l\tilde{{{\Gamma }}}({l}^{2}+l-2))\\ &-2{j}_{l+1}(\eta )({\eta }^{2}-l(\tilde{{{\Gamma }}}+2)({l}^{2}+l-2)).\end{array}$$

**Fig. 7:**
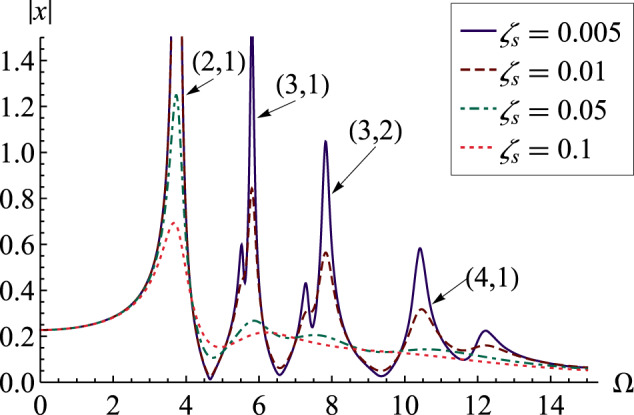
Frequency-response diagram for Kelving–Voigt material. The plot shows the amplitude of drop motion ∣*x*∣ against the driving frequency Ω as it depends upon *ζ*_*s*_, with Γ = 1. Resonance peaks for the (*l*, *s*)=(2, 1),(3, 1),(3, 2), and (4, 1) modes are shown.

For a given rheology $\tilde{\mu }$, Eq. (16) gives a complex radial displacement whose magnitude ∣*x*∣ provides the amplitude of drop motion for a given driving frequency. A typical frequency-response diagram is shown in Fig. 7, which plots the amplitude ∣*x*∣ against driving frequency Ω for the Kelvin–Voigt model, as it depends upon *ζ*_*s*_. Each local maximum corresponds to a resonance peak where the driving frequency is the same as the natural frequency of the drop, i.e., Ω = *ξ*. As *ζ*_*s*_ increases, the amplitude of the resonance peaks decreases consistent with viscous damping. Higher-order modes are damped at a higher rate and for *ζ*_*s*_ = 0.1, the resonance peaks for the *l* > 2 modes have nearly disappeared. This has been previously observed in the oscillation of sessile gel drops^52^. Recall in Fig. 4 that this disappearance of the resonance peak at a critical *ζ*_*s*_ was shown for a fractional Kelvin model. Comparing Fig. 4 with our analysis here on forced vibration shows that beyond the critical *ζ*_*s*_ pointed out by the red cross, oscillations predicted by the fractional model do not result in a physically observable resonance peak.

In general, the drop response is complex and admits a real and imaginary part. As such, one can define a phase angle as

17 $$\alpha =\arctan \left|\frac{\,{{\mbox{Im}}}\,[x]}{\,{{\mbox{Re}}}\,[x]}\right|.$$

**Fig. 8:**
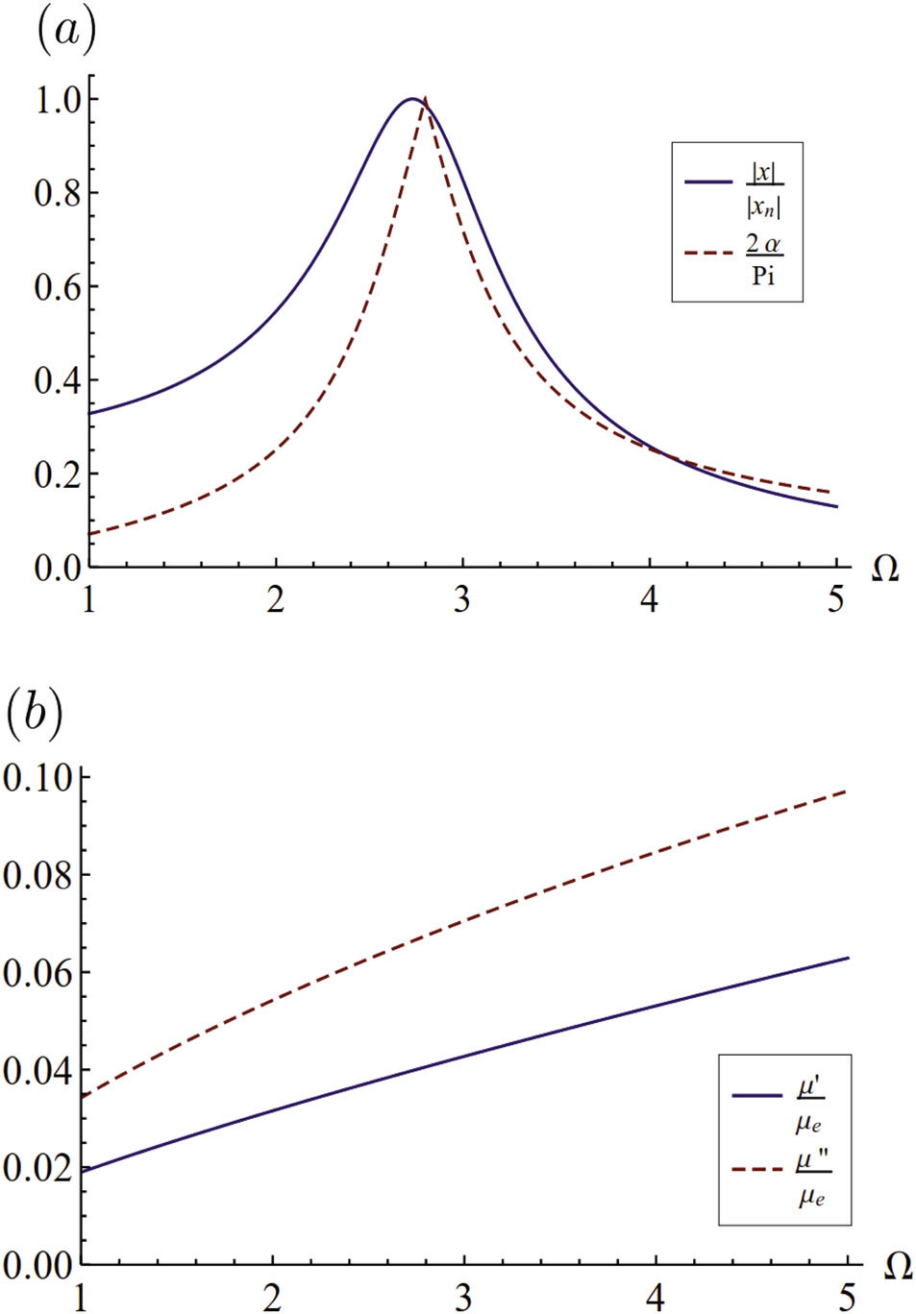
Drop response and complex modulus, as functions of driving frequency, for fractional Maxwell material. **a** Amplitude ratio $\frac{| x| }{| {x}_{n}| }$ and phase angle 2*α*/*π* against driving frequency Ω with Γ = 0.1, *ζ*_*f*_ = 0.01. Here ∣*x*_*n*_∣ is the amplitude at the resonance frequency. **b** Dimensionless storage $\left(\frac{\mu ^{\prime} }{{\mu }_{e}}\right)$ and loss $\left(\frac{{\mu }^{^{\prime\prime} }}{{\mu }_{e}}\right)$ moduli for the same parameters.

That is, the motion can be completely determined from knowledge of the frequency response and phase angle. This is shown in Fig. 8a for the *l* = 2 mode of a fractional Maxwell material. The storage and loss moduli for these frequencies are shown as scaled with *μ*_*e*_ in Fig. 8b.

#### Drop-vibration rheometer

The experimentally observed frequency response of a drop has been used to infer both the surface tension and viscosity of Newtonian fluids with most prior work done with the *l* = 2 fundamental mode. We briefly describe the approach of Hosseinzadeh and Holt^53^, who have used the generic frequency response for a damped oscillator,

18 $$\frac{x}{{x}_{o}}=\frac{1}{\sqrt{{\left(1-{\left(\frac{\omega }{{\omega }_{n}}\right)}^{2}\right)}^{2}+\frac{2{{\Delta }}f{\omega }^{2}}{{\omega }_{n}^{3}}}},$$

to fit to their experimental data with *ω*_*n*_, Δ*f* fitting parameters. Here *x*_*o*_ is the driving amplitude, *ω*_*n*_ is the resonance frequency related to ${{{\rm{Re}}}}[\xi ]$, and Δ*f* related to the bandwidth of the resonance peak and effectively the decay rate ${{{\rm{Im}}}}[\xi ]$. For a Newtonian fluid, the resonance frequency has been given by^4^

19 $${\omega }_{n}^{2}=\frac{8\sigma }{\rho {R}^{3}},$$

and the spectral width by^24,25^

20 $${{\Delta }}f=\frac{5\nu }{{R}^{2}},$$

with *ν* the kinematic viscosity. Shao et al.^35^ have taken a similar approach for gel drops but included the added effect of elasticity.

**Fig. 9:**
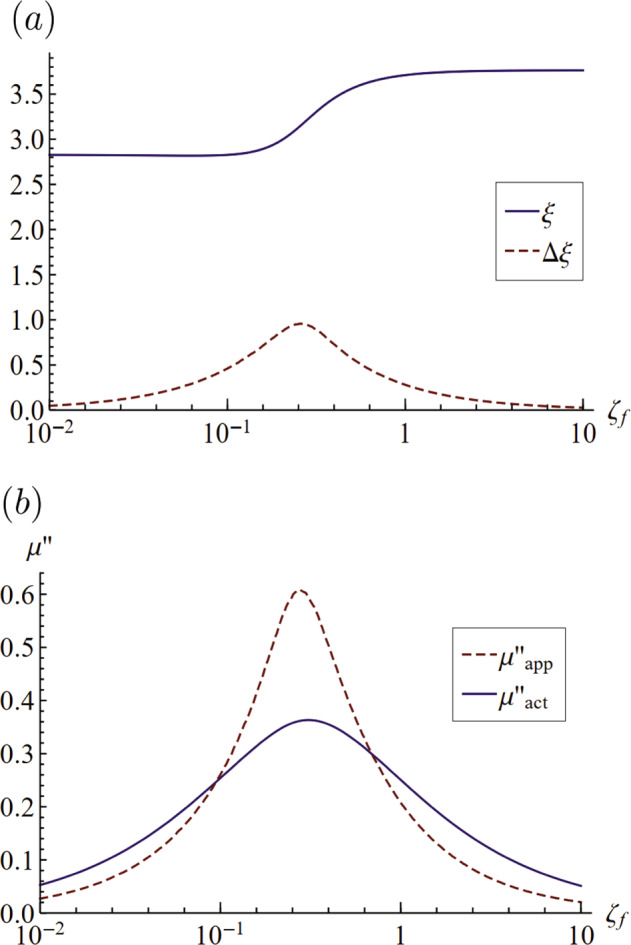
Error in rheology measurement using a model for Newtonian fluid. **a** Resonance frequency *ξ* and spectral width Δ*ξ* against the Deborah number *ζ*_*f*_ for fractional Maxwell model with Γ = 1, *n* = 0.8. **b** The actual loss modulus ${\mu }_{\,{{\rm{act}}}\,}^{{\prime\prime} }$ for this set of parameters compared against the approximate loss modulus ${\mu }_{\,{{\rm{app}}}\,}^{{\prime\prime} }$ obtained by Lamb’s equation of viscous decay.

Unfortunately, this approach only works for materials with frequency-independent material properties, i.e., not for viscoelastic materials. To be more specific, Eq. (20) can not be used to fit to the response diagram to measure viscosity accurately for viscoelastic materials. This is illustrated in Fig. 9a, where we find the resonance peak *ξ* and spectral width Δ*ξ* for a fractional Maxwell material (*n* = 0.8) as it depends upon *ζ*_*f*_. Since we are using a known rheology, the values of $\mu ^{\prime}$ and *μ″* are known and we compare this with Eq. (20) by taking *μ″* = *ν*/*ω*, as shown in Fig. 9b. Here we see that the predicted values are different from the known rheology, suggesting that it is not possible to use this particular approach to infer material properties. However, given that our model is applicable for any rheology, we expect that it would be possible to infer the rheology of a forced viscoelastic drop from its frequency response *and* phase angle over the appropriate range of driving frequencies. This would allow one to measure $\mu ^{\prime}$ and *μ″* as two fitting parameters for these two sets of data. For example, if we know the frequency and amplitude diagram over a frequency spectrum as shown in Fig. 8a, it would be possible to extract $\mu ^{\prime}$ and *μ″* at each frequency Ω in Fig. 8b. This would be done by using the values of ∣*x*∣ and *α* in the real and imaginary parts of Eq. (16) and solve them as two equations with unknown variables $\mu ^{\prime}$ and *μ″* at each different frequencies.

Recently, Temperton et al.^38^ have used a modified version of Eq. (20) to measure the storage and loss modulus at the resonance frequency. This model assumes a flat, semi-infinite surface ignoring the surface curvature inherent in a sphere^33^. This method is developed without needing to know the phase angle and enables measurement of the rheology, which differs slightly from that obtained with a traditional cone-plate rheometer. We note that our model could be used to improve on this particular method.

## Discussion

We have performed a theoretical analysis of the free and forced oscillations of a soft viscoelastic drop, deriving the dispersion relationship for a soft viscoelastic material with arbitrary rheology. We illustrate the utility of our solution by computing (1) the natural frequencies for the free-oscillation case and (2) the frequency-response diagram for the forced-oscillation case, for the classical Kelvin–Voigt and Maxwell models of viscoelasticity, as well as their fractional variants. For these materials, the oscillations depend upon two dimensionless quantities: (1) the elastocapillary number and (2) the Deborah number. For both Kelvin–Voigt and Maxwell models, the motion changes from underdamped to overdamped as viscosity increases and we map out this boundary in the parameter space. However, for the fractional models, we find that although viscosity still reduces oscillation, no overdamped motions exist, i.e., there is always a nonzero oscillation frequency associated with the large viscous decay rates.

Our analysis of the forced-oscillation problem could be used as a “drop vibration rheometer” by combining theoretical predictions with experimental observations. This is in a similar spirit to other work on Newtonian fluids^54^ and soft purely elastic gels^39,55^. Such a drop-vibration rheometer could be a particularly useful noncontact method for rheological measurement that has implications in monitoring blood-clot and blood-cell diseases^53,54^. Last, we note that our current analysis is limited to small-amplitude oscillation of viscoelastic drops. Large deformations in soft viscoelastic materials can often give rise to nonlinear behaviors such as strain stiffening and shear thickening. Diagnostic methods for such materials have been the focus of recent studies^56,57^. To the best of our knowledge, no attempts have yet been made to probe the nonlinear response of viscoelastic drops. Future works could focus on developing a theoretical basis for nonlinearities in drop oscillation, as well as investigating other non-Newtonian fluids, e.g., yield-stress fluids.

## Supplementary information

Supplementary Information

Reporting Summary

## Data Availability

All data presented in the plots are available from the authors upon reasonable request.

## Code availability

The code used to generate the plots is available from the authors upon reasonable request.
