# Supplementary material for: Oscillations of a soft viscoelastic drop
Source: NPJ Microgravity. 2021 Nov 2;7:42. doi: 10.1038/s41526-021-00169-1 (PMC8563899; doi:10.1038/s41526-021-00169-1)
Supplement: Supplementary file 1 — Supplementary Information [file 41526_2021_169_MOESM1_ESM.pdf]

Supplemental Information

Oscillations of a soft viscoelastic drop

Applying the general solutions from Eq. (12) to Eq. (8) give,

$$\begin{aligned}
&-P_o + A_{lm}F_{11} + C_{lm}F_{12} \\
&\qquad = \frac{\tilde{\Gamma}}{2}(2 - l - l^2)(A_{lm}G_1 + C_{lm}G_2) \\
&A_{lm}F_{21} + C_{lm}F_{22} = 0 \\
&B_{lm}F_{31} = 0
\end{aligned}$$

with,

$$\begin{aligned}
F_{11} &= l^2 - l - \frac{\xi^2\tilde{\Gamma}}{2} \\
F_{12} &= l(l+1)((l-1)j_l(\xi\sqrt{\tilde{\Gamma}}) - \xi j_{l+1}(\xi\sqrt{\tilde{\Gamma}})) \\
G_1 &= lR^l \\
G_2 &= l(l+1)j_l(\xi\sqrt{\tilde{\Gamma}}) \\
F_{21} &= 2(l-1)R^l \\
F_{22} &= (2l^2 - 2 - \xi^2\tilde{\Gamma})j_l(\xi\sqrt{\tilde{\Gamma}}) + 2\xi\sqrt{\tilde{\Gamma}}j_{l+1}(\xi\sqrt{\tilde{\Gamma}}) \\
F_{31} &= (l-1)j_l(\xi\sqrt{\tilde{\Gamma}}) - \xi\sqrt{\tilde{\Gamma}}j_{l+1}(\xi\sqrt{\tilde{\Gamma}})
\end{aligned}$$

The external pressure $P_o = 0$ for free vibration and $P_o = P_d e^{i\omega t}$ for forced vibration.

The dispersion relation for the torsional modes is given by,

$$F_{31} = 0$$
